# Insights into Mechanisms of Damage Recognition and Catalysis by APE1-like Enzymes

**DOI:** 10.3390/ijms23084361

**Published:** 2022-04-14

**Authors:** Anatoly A. Bulygin, Olga S. Fedorova, Nikita A. Kuznetsov

**Affiliations:** 1 Institute of Chemical Biology and Fundamental Medicine, SB RAS, 630090 Novosibirsk, Russia; skytolya@ya.ru; 2 Department of Natural Sciences, Novosibirsk State University, 630090 Novosibirsk, Russia

**Keywords:** base excision repair, apurinic/apyrimidinic endonuclease, conformational dynamics, active-site plasticity, damaged nucleotide, nucleotide eversion, nucleotide incision activity

## Abstract

Apurinic/apyrimidinic (AP) endonucleases are the key DNA repair enzymes in the base excision repair (BER) pathway, and are responsible for hydrolyzing phosphodiester bonds on the 5′ side of an AP site. The enzymes can recognize not only AP sites but also some types of damaged bases, such as 1,*N*^6^-ethenoadenosine, α-adenosine, and 5,6-dihydrouridine. Here, to elucidate the mechanism underlying such a broad substrate specificity as that of AP endonucleases, we performed a computational study of four homologous APE1-like endonucleases: insect (*Drosophila melanogaster*) Rrp1, amphibian (*Xenopus laevis*) APE1 (xAPE1), fish (*Danio rerio*) APE1 (zAPE1), and human APE1 (hAPE1). The contact between the amino acid residues of the active site of each homologous APE1-like enzyme and the set of damaged DNA substrates was analyzed. A comparison of molecular dynamic simulation data with the known catalytic efficiency of these enzymes allowed us to gain a deep insight into the differences in the efficiency of the cleavage of various damaged nucleotides. The obtained data support that the amino acid residues within the “damage recognition” loop containing residues Asn222–Ala230 significantly affect the catalytic-complex formation. Moreover, every damaged nucleotide has its unique position and a specific set of interactions with the amino acid residues of the active site.

## 1. Introduction

The integrity of DNA in the cell is preserved by a complex repair system that implements the recognition and removal of DNA damage and the restoration of normal DNA structure [1]. Base excision repair is one of the pathways of DNA repair intended to eliminate non-bulky lesions. Base excision repair is usually initiated by damage-specific DNA glycosylases [2,3] that recognize damaged bases and generate apurinic/apyrimidinic (AP) sites in their place; alternatively, AP sites can be generated directly by spontaneous hydrolysis of the *N*-glycosidic bond. Then, the AP endonuclease hydrolyzes the phosphodiester bond on the 5′ side of the abasic nucleotide [4], followed by the DNA-template-directed incorporation of a nucleotide by DNA polymerase and DNA ligation. It was found that DNA repair can be initiated directly by AP endonucleases in the nucleotide incision repair (NIR) pathway; this can occur when an enzyme directly recognizes and catalyzes the cleavage of DNA on the 5′ side of various specific non-bulky lesions, such as the relatively common naturally occurring 5,6-dihydrouridine (DHU); α-anomers of nucleotides (e.g., αA); 1,*N*^6^-ethenoadenosine (εA); 2′-deoxyuridine (U); and other modified residues [5,6,7,8,9,10,11].

**Figure 1:**
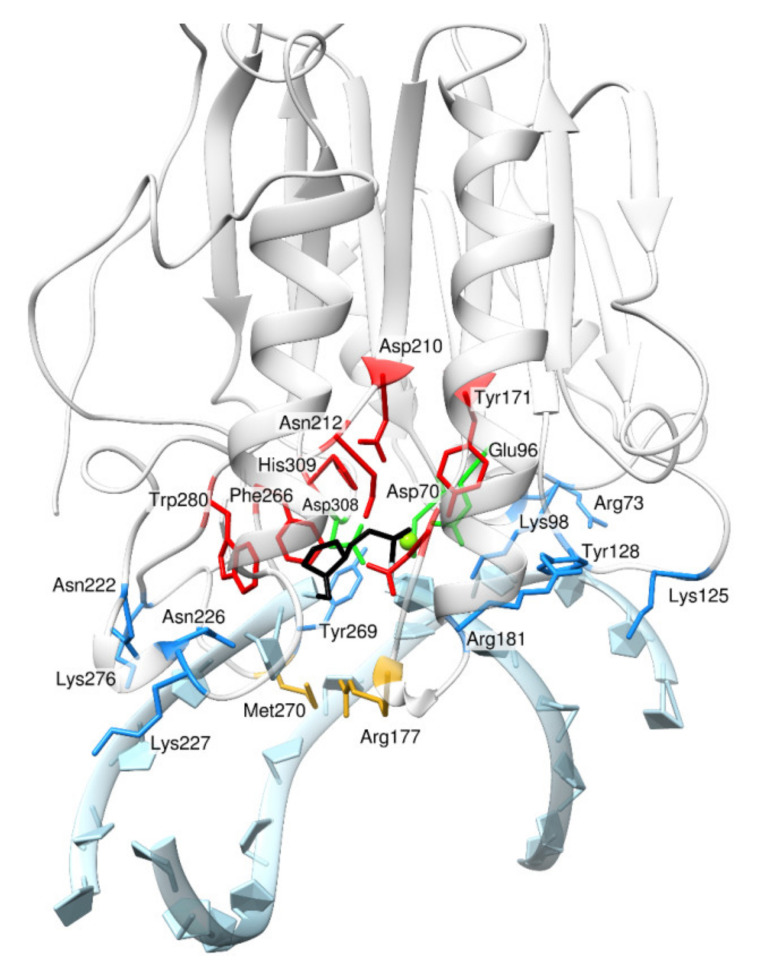
The model of hAPE1 complexed with DNA containing an F-site [29]. Active-site amino acid residues Tyr171, Asn174, Asp210, Asn212, Phe266, Trp280, and His309 are red; Mg^2+^-coordinating residues Asp70, Glu96, and Asp308 are green; DNA-binding–site residues Arg73, Lys98, Lys125, Tyr128, Arg181, Asn222, Asn226, Lys227, Lys276, and Tyr269 are blue; and intercalating residues Met270 and Arg177 are yellow. The F-site is black.

Human AP endonuclease 1 (hAPE1) is one of the most studied AP endonucleases and a major human enzyme from this family. Currently, there are more than 50 crystallized structures of APE1-like enzymes deposited in the PDB [12,13,14,15,16,17,18], and tens of publications with kinetic [19,20,21,22,23] and a mutational [24,25,26,27,28] data available. Still, there is no three-dimensional structure of any AP endonuclease in complex with a DNA substrate containing any damaged base that is recognized as a substrate in the NIR pathway. Crystal structures of the complex of hAPE1 bound to an abasic DNA containing an F-site—tetrahydrofuran-containing AP site, a nonreactive analog of a natural AP site—revealed a set of amino acid residues that are in contact with the DNA duplex and with the damaged nucleotide in particular (Figure 1). The DNA-binding site of hAPE1 consists of a number of residues, including Lys98, Tyr128, Arg181, Asn222, Asn226, and Tyr269, which form hydrogen bonds and electrostatic contact with DNA. Structural data also showed that for a catalytic complex to arise, the F-site needs to be everted from the DNA helix into the active site. The extrahelical state of the damage is stabilized by Arg177 and Met270, which are placed into the void left by the damaged nucleotide after its eversion. The volume of the active site is confined by Asn174, Gly231, Phe266, Trp280, and Leu282. The set of the residues required for the catalytic reaction includes Tyr171, Asp210, Asn212, His309, and three Mg^2+^-coordinating residues: Asp70, Glu96, and Asp308.

Studies on the kinetics of the hAPE1 mechanism of action have shown that it involves two steps: the formation of the initial enzyme–substrate complex and the flipping of the damaged nucleotide into the enzyme active site [21]. Later, it was found that the rate of the second step is highly dependent on the type of damage: the processing time of the F-site is ~1 s [26,27,30], whereas such damaged nucleotides as εA, αA, and DHU are processed for up to 1000 s [31,32]. In our previous study involving molecular dynamics (MD) simulations, we demonstrated that some damaged bases form specific H-bonds with the enzyme [29]. Thus, we can hypothesize that the ability of a damaged nucleotide to flip into the active site depends on two factors: steric interactions between the lesion and a protein during the flipping, and “pulling” forces between the damaged base and protein realized through the emergence of temporary H-bonds, such as Asn229 N^δ2^–DHU O2 and Asn212 N^δ2^–αA N3. In the same work [29], it was found that there are interactions of this type that arise between the protein loop containing Asn229/Thr233/Glu236 and a damaged base. This loop appears to be flexible and amenable to reshaping by the damaged base, thereby leading to an increase in the active-site pocket volume. Nevertheless, there are other important questions: does the steric hindrance have the strongest impact on the damage flipping, and when, exactly, does an obstacle appear?

**Figure 2:**
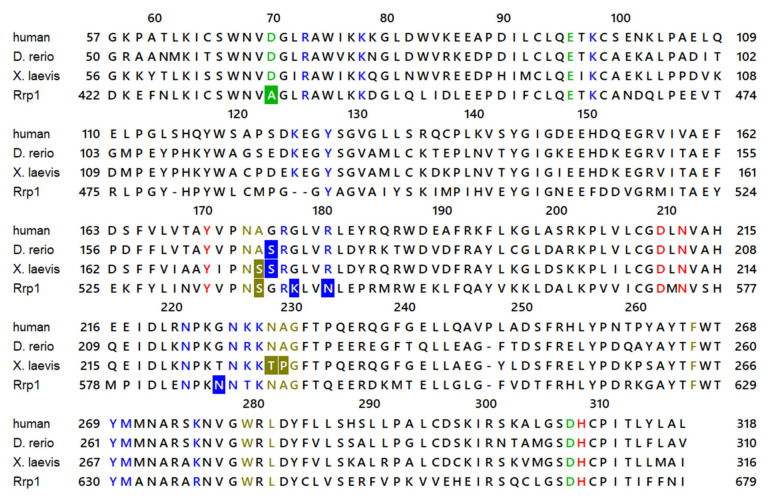
The alignment of the catalytic domain of four APE1-like enzyme sequences. Mg^2+^-coordinating residues are highlighted in green, catalytic residues are red, DNA-binding residues are blue, and active-site pocket residues are yellow. Substitutions that may be important have a colored background. Rrp1 contains one important substitution—D70A—that is known to reduce hAPE1 efficiency, and four alterations (Lys125-, Gly178Lys, Arg181Asn, and Gly225Asn) in the DNA-binding interface. xAPE1 has three substitutions (Ala175Ser, Asn229Thr, and Ala230Pro) at the periphery of the active site.

We recently reported the results of a study on four homologous APE1-like endonucleases with strong NIR activity: insect (*Drosophila melanogaster*) Rrp1, amphibian (*Xenopus laevis*) APE1 (xAPE1), fish (*Danio rerio)* APE1 (zAPE1), and hAPE1 [33]. Our data revealed that Rrp1 is far less efficient in the processing of the F-site and is the only enzyme (among the four) not able to process αA, whereas zAPE1 and xAPE1 differ in certain kinetic parameters but not in overall efficacy. A comparison of the sequences and structures of the four proteins indicated that Rrp1 bears several substitutions that reduce the total identity of the amino acid sequence of the catalytic domain, in comparison with hAPE1, to 50% (Figure 2). Despite such a difference in total amino acid sequences, the most important catalytic and substrate-binding amino acid residues are identical, except for substitutions Gly178Lys, Arg181Asn, and Gly225Asn; the deletion of Lys125 (amino acid numbering corresponds to hAPE1 sequence) in the DNA-binding site; and substitution Asp70Ala at a Mg^2+^-coordinating site. The first XYZ is known to decrease binding affinity for a DNA product [17], and the second one affects AP endonuclease and NIR activity in hAPE1 [34]. Other than that, both zAPE1 and xAPE1 manifested 66–67% identity with hAPE1, but only xAPE1 has substitutions Ala175Ser, Asn229Thr, and Ala230Pro in the vicinity of the active site. Altogether, these alterations may have a strong impact on the enzyme’s efficiency.

The main aim of this study was to obtain molecular models of the structure of enzyme–substrate complexes of the four homologous APE1-like endonucleases with DNA duplexes containing a damaged nucleotide: an F-site, εA, αA, or DHU. A comparison of MD simulation data with known catalytic efficiency of these enzymes, as determined previously [33], allowed us to gain a deep insight into the mechanism of damage recognition, to elucidate differences in the efficiency of cleavage of various damaged nucleotides, and to find new distinctive features of APE1-like enzymes.

## 2. Results and Discussion

Structural models of complexes of all the analyzed APE1-like enzymes with abasic DNA were built on the basis of the X-ray structure of an hAPE1–DNA complex consisting of the protein and an 11 bp DNA duplex containing an F-site (Protein Data Bank (PDB) ID 4IEM) [18]. It should be noted that hAPE1 has a rigid protein core, and DNA binding leads to negligible structural rearrangements. In various hAPE1 structures, the differences in the rotamers of the side chains of some peripheral residues and some residues of a DNA-binding cleft were found, which were typically negligible after MD equilibration. In the case of zAPE1, the X-ray structure of the free protein (PDB ID 2O3C, [35]) was used to build a model of the zAPE1–DNA complex. The protein structures of the AP endonucleases xAPE1 and Rrp1 were obtained through homology modeling using the hAPE1 structure as a template; this analysis, respectively, revealed 67% and 50% of the identity of amino acid sequences of the catalytic domain. These protein models were employed to build models of the enzyme–DNA complexes, as described in the Materials and Methods section.

**Figure 3:**
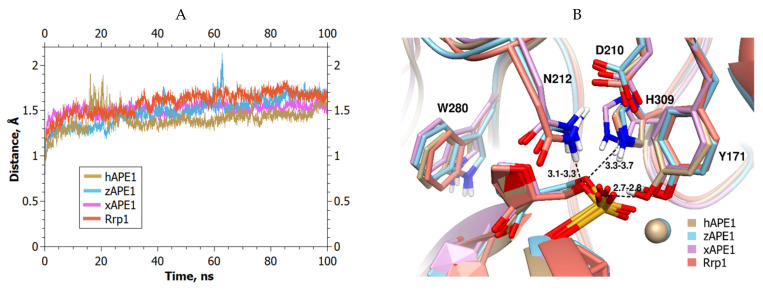
MD trajectories and structures of the complexes of APE1-like endonucleases with abasic DNA: (**A**) Changes in RMSD in the MD trajectories for the analyzed complexes of APE1-like enzymes with DNA containing the F-site; (**B**) structural models of enzyme–substrate complexes (hAPE1 is sand-colored, zAPE1 is cyan, xAPE1 is magenta, and Rrp1 is red). F-site and 5′- phosphate group in the active sites of APE1-like enzymes are shown. The figure also presents ranges of the average lengths (Å) of H-bonds between His309 N^ε2^ and O5′, between Asn212 N^δ2^ and O5′, and between Tyr171 O^η^ and O2P for different complexes.

First of all, equilibrated for 100–200 ns, MD trajectories were generated for all enzyme–DNA complexes to obtain conformationally optimized structural models (Figure 3A). These MD trajectories were utilized to determine the catalytically competent positions of an abasic nucleotide in the active site by evaluating the relative positions of the damaged nucleotide and of the active-site amino acid residues. The changes in the distances between the atoms of the damaged nucleotide and the atoms of catalytic amino acid residues during the simulation helped to calculate the average distances, which were used for a comparison of the investigated APE1-like enzymes. An analysis of MD trajectories of all the investigated enzymes revealed no significant differences in the distances between catalytic residues and the F-site in DNA, indicating good quality of all the obtained structural models of the enzyme–substrate complexes (Figure 3B).

In all the models, the phosphate group of the F-site formed stable H-bonds with Tyr171 O^η^ and Asn212 N^δ2^, with an average length of 2.7 and 3.2 Å, respectively. The scissile phosphate group engaged in a strong interaction with Mg^2+^ (the distance between oxygen O1P and Mg^2+^ was 1.8 Å) in all the obtained complexes. The average distance between the O5′ atom of the F-site and the Asp210 O^δ1^ atom was stabilized at 4.4 Å, and the average distance between the O5′ atom of the F-site and His309 N^ε2^ varied from 3.3 Å in xAPE1 to 3.7 Å in Rrp1.

Equilibrated structures of the complex of enzymes with F-site-containing DNA were used to create structural models of the complex with DNA containing DHU, αA, or εA, as described earlier for hAPE1 [29]. Each complex was then equilibrated for 100–200 ns at 300 K (room temperature) to determine the average distances between key amino acid residues and a damaged nucleotide in the active site.

**Figure 4:**
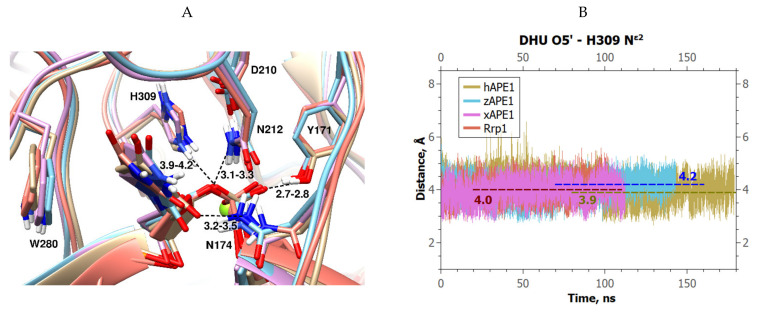
MD trajectories and structures of the complexes of APE1-like endonucleases with DHU-containing DNA: (**A**) Structural models of enzyme–substrate complexes with average lengths of H-bonds (hAPE1 is sand-colored, zAPE1 is cyan, xAPE1 is magenta, and Rrp1 is red). The average lengths of H-bonds between His309 N^ε2^ and DHU O5′, between Asn212 N^δ2^ and DHU O5′, between Asn174 N^δ2^ and DHU O2, and between Tyr171 O^η^ and DHU O2P are given; (**B**) Changes in DHU O5′–His309 N^ε2^ distances in different complexes throughout the MD trajectories for the analyzed complexes of APE1-like enzymes with DHU-containing DNA.

It turned out that the position of the DHU base in the active sites was similar among all the enzymes under study, and close to the position reported earlier for hAPE1 (Figure 4A). The phosphate group of DHU in all four complexes also entered into stable H-bonds with Tyr171 O^η^ (bond length 2.7–2.8 Å) and Asn212 N^δ2^ (3.1–3.3 Å). The average distance between DHU O5′ and Asp210 O^δ1^ was found to be 4.5 Å, i.e., very similar to the value of 4.4 Å shown by the F-site-containing DNA (Figure 3). The distance between the O1P oxygen and Mg^2+^ proved to be very stable, too (2.0 Å on average), in all the obtained complexes. Nevertheless, the average distance between DHU O5′ and His309 N^ε2^ was slightly greater than that in complexes with the F-site; it varied from 3.9 to 4.2 Å among the different enzymes (Figure 4B). Notably, in all the complexes, the DHU base formed a stable H-bond (3.2–3.5 Å) between the O2 atom and Asn174 N^δ2^.

Previously, regarding hAPE1, it was reported [29] that the binding of a damaged nucleotide in the active site of this enzyme induces significant conformational reorganization of the “damage recognition” loop containing residues Asn229–Thr233, thereby resulting in a loss of the H-bond between Thr233 O^γ1^ and Glu236 O^ε1^ and in enlargement of the inner volume of the damaged-base-binding pocket. It was suggested that destabilization of the α-helix containing Thr233 and Glu236 via a loss of the interaction between these residues increased the plasticity of the damaged-nucleotide-binding pocket and the ability to accommodate structurally different damaged nucleotides.

**Figure 5:**
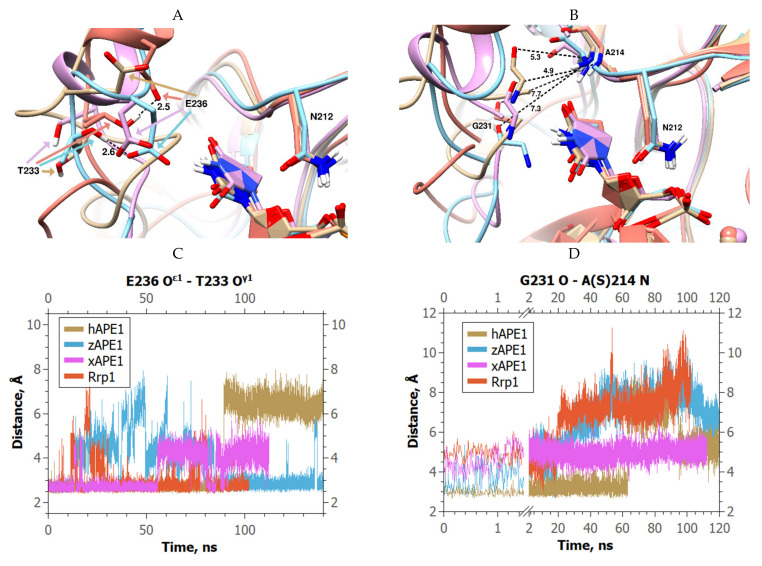
Changes in the damage-recognition loop in the complexes of the investigated APE1-like enzymes with DHU-containing DNA (hAPE1 is sand-colored, zAPE1 is cyan, xAPE1 is magenta, and Rrp1 is red): (**A**) Changes in the Asn229–Thr233 residues in the complexes of the four enzymes with DHU-containing DNA; (**B**) alternations of the DHU O5′–His309 N^ε2^ distance for different enzymes. Black dashed lines indicate unsevered H-bonds; overall changes in Thr233 O^γ1^–Glu236 O^ε1^ (**C**) and Gly231 O–Ala214 N (**D**) distances in different complexes throughout the MD trajectories.

Our findings revealed that the temporary loss of this H-bond took place in each tested model containing DHU (Figure 5A). Further investigation of the models uncovered a more relevant change in the structures. We found that the H-bond between Gly231 O and Ala214 N was lost permanently in every trajectory, thus facilitating a movement of the damage-recognition loop away from the damaged base (Figure 5B). The overall dynamics of distances Thr233 O^γ1^–Glu236 O^ε1^ and Gly231 O–Ala214 N in the complexes of enzymes with DHU-containing DNA are presented in Figure 5C,D. Notably, both H-bonds (Thr233 O^γ1^–Glu236 O^ε1^ and Gly231 O–Ala214 N) were stable in the MD trajectories of the complexes of all enzymes with F-site-containing DNA.

**Figure 6:**
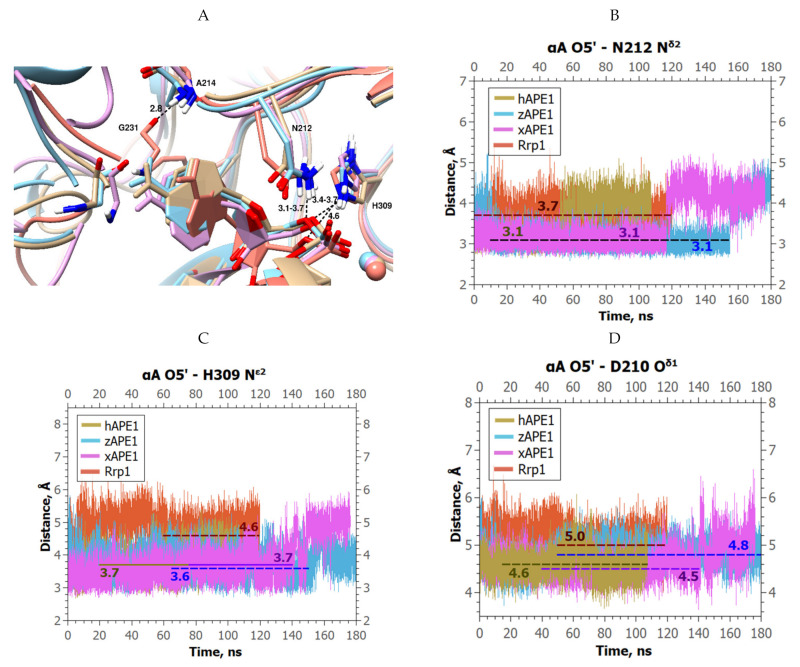
MD trajectories and structures of the complexes of APE1-like endonucleases with αA-containing DNA (hAPE1 is sand-colored, zAPE1 is cyan, xAPE1 is magenta, and Rrp1 is red): (**A**) Structural models of enzyme–substrate complexes and average lengths of H-bonds. The average lengths of H-bonds between His309 N^ε2^ and αA O5′, between Asn212 N^δ2^ and αA O5′, and between Gly231 O and Ala214 N are presented; alterations of (**B**) αA O5′–Asn212 N^δ2^, (**C**) αA O5′–His309 N^ε2^, and (**D**) αA O5′–Asp210 O^δ1^ distances throughout the MD trajectories of the analyzed APE1-like enzymes in complex with αA-containing DNA.

Analysis of the complexes of hAPE1, zAPE1, and xAPE1 with DNA containing αA revealed that in the course of the MD simulation, the damaged base turned by 70–90° around the *N*-glycosidic bond toward the Asn212 sidechain; thus, it yielded an increase in the αA O5′–Asn212 N^δ2^ distance from 3.1 to ~4.4 Å, revealing the loss of the bond (Figure 6A,B). On the contrary, in the MD trajectory of Rrp1, the average αA O5′–Asn212 N^δ2^ distance proved to be stable (3.6–3.8 Å) and only slightly increased relative to this value in the complex with the F-site (3.1–3.3 Å, Figure 3B).

Furthermore, a slight increase in distance αA O5′–His309 N^ε2^ (3.6–3.8 Å) was registered in the initial part of the MD trajectory for hAPE1, zAPE1, and xAPE1 when compared with F-site-containing DNA (Figure 6C). In the case of hAPE1 and xAPE1, this distance additionally grew to 4.2 and 5.0 Å, respectively, in the final part of the trajectory, meaning a complete loss of this contact. By contrast, in the case of Rrp1, the αA O5′–His309 N^ε2^ distance turned out to be relatively long and stable (4.5–4.6 Å). Similar behavior was documented for another catalytic residue, Asp210 (Figure 6D): in the xAPE1 simulation, the distance between αA O5′ and Asp210 O^δ1^ increased from 4.5 to 5.0 Å at the end of the trajectory, but remained relatively stable in the other three complexes, being notably greater in the complex involving Rrp1: 5.0 instead of 4.5–4.6 Å as in the other cases.

The analysis of all the trajectories showed that sudden increases in the distances between different active-site residues and αA’s atoms could be attributed to the participation of a water molecule that entered the active site. On the other hand, the active site of Rrp1 was the only one not affected by water molecules; accordingly, all the mentioned distances remained stable. Moreover, in all the trajectories with the F-site and DHU described above, there was no water in the active site either; hence, no sudden jumps in distance were observed. It is interesting to note that the X-ray structures of hAPE1-DNA complexes confirmed the presence of a water molecule in the active site, which is necessary for the catalytic hydrolysis of DNA. Therefore, water molecule movement in the active site and the associated extensive conformational changes in amino acid residues could both be important factors affecting enzyme activity toward different damaged substrates.

**Figure 7:**
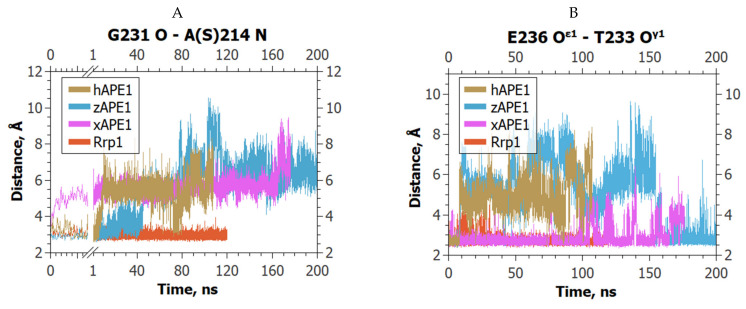
The dynamics of (**A**) Gly231 O–Ala214 N and (**B**) Thr233 O^γ1^–Glu236 O^ε1^ distances in the complexes of the APE1-like enzymes with αA-containing DNA.

Major differences between the studied enzymes were observed in the behavior of the damage-recognition loop. In the MD trajectories, the average lengths of the H-bonds Thr233 O^γ1^–Glu236 O^ε1^ and Gly231 O–Ala214 N were stable only in the case of Rrp1 (Figure 7). All these alterations caused significantly different positioning of αA in the active site of Rrp1 (Figure 6A).

**Figure 8:**
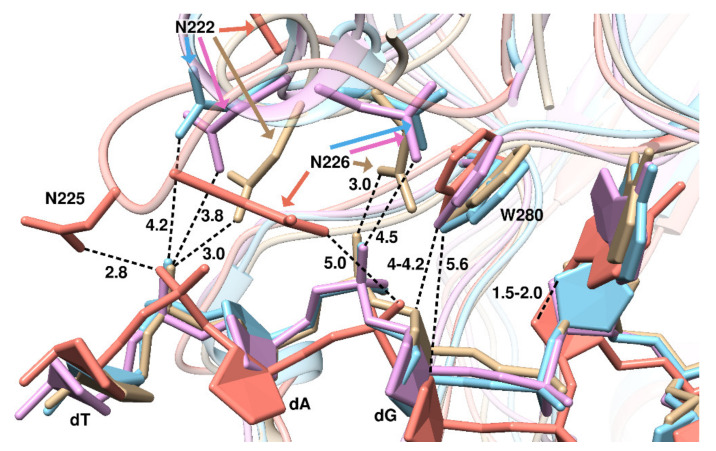
The position of αA, and the structure of the Asn222–Ala230 loop in different models (hAPE1 is sand-colored, zAPE1 is cyan, xAPE1 is magenta, and Rrp1 is red). To demonstrate the shift of the DNA backbone in the complex with Rrp1 relative to the others, the distance between two αA C2′ atoms of the models involving zAPE1 and Rrp1 is shown (1.5–2.0 Å), as are the distances between hAPE1 Trp280 N^ε1^ and C3′ atoms of dG in the models involving hAPE1 (4.0–4.2 Å) and Rrp1 (5.6 Å).

To understand the reasons for the differences in αA positions between the Rrp1 complex and the complexes of the other enzymes under study, we examined the vicinity of the damaged nucleotide in all the complexes. We noticed a significant deviation in the complex with Rrp1 at the positions of the deoxyribose of αA and its neighboring (on the 3′ side) dG’s backbone (Figure 8): 2′-deoxyribose C1′, C2′, and C3′ atoms of αA were shifted from the DNA-binding cleft by 1.5–2.0 Å relative to the corresponding 2′-deoxyriboses in the cases of the other three enzymes. Additionally, the atoms of dG located on the 3′ side of αA were at least 1.5 Å further from the amino acid residues of the active site. For example, the distance between the C3′ of dG and the N^ε1^ of Trp280, whose spatial position is highly conserved, rose from ~4.0 to ~5.6 Å. Taken together, these observations suggest that Rrp1 applies a weaker pulling force on DNA; thus, it is unable to place the DNA backbone deeply into the DNA-binding cleft, thereby leading to the damaged nucleotide’s position, which is less favorable for catalysis.

The phosphate groups of the next two nucleotides in the direction of the 3′ side of αA (dA and dT) interacted with Asn226 and Asn222, respectively, in the complexes with APE1s; the distances between the N^δ2^ of asparagines and the nonbridging oxygens of phosphates were both 3.0 Å for hAPE1, and varied from 3.8 to 4.5 Å in zAPE1 and xAPE1. In Rrp1, Asn226 also periodically interacted with the phosphate group of dT (the distance jumped between 3.0 and 5.0 Å) but was located on the other side of it. Instead of Asn222 (which moved far away from DNA), Asn225—the analog of Gly225 in APE1—interacted with dA, also on the other side.

**Figure 9:**
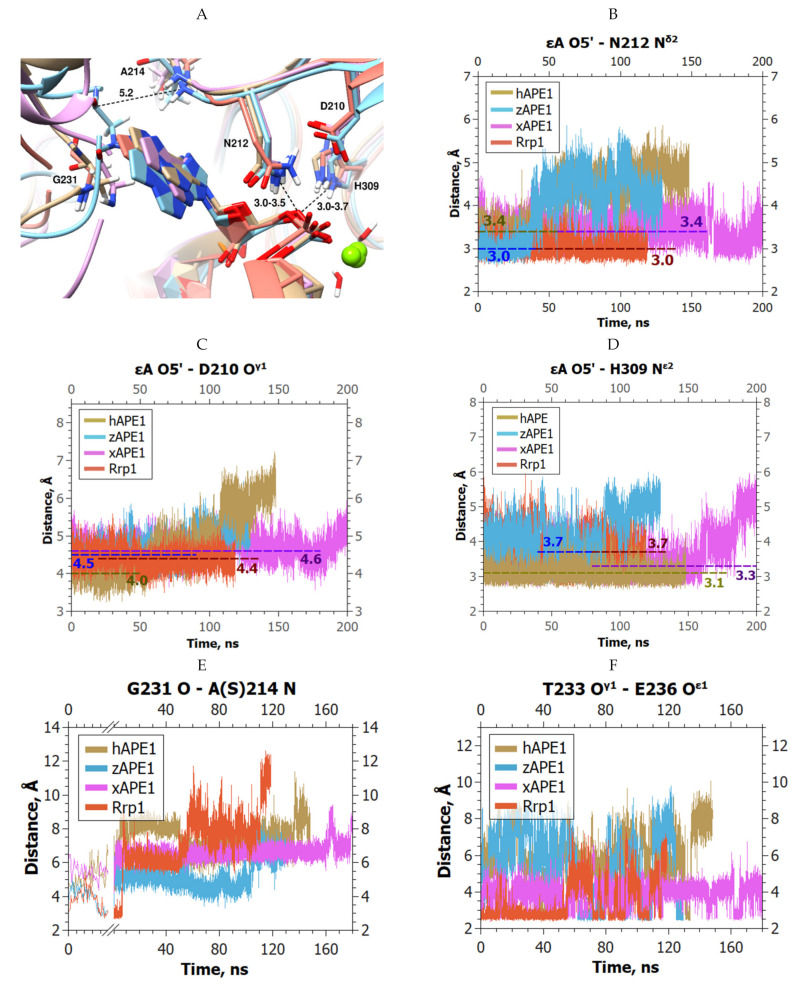

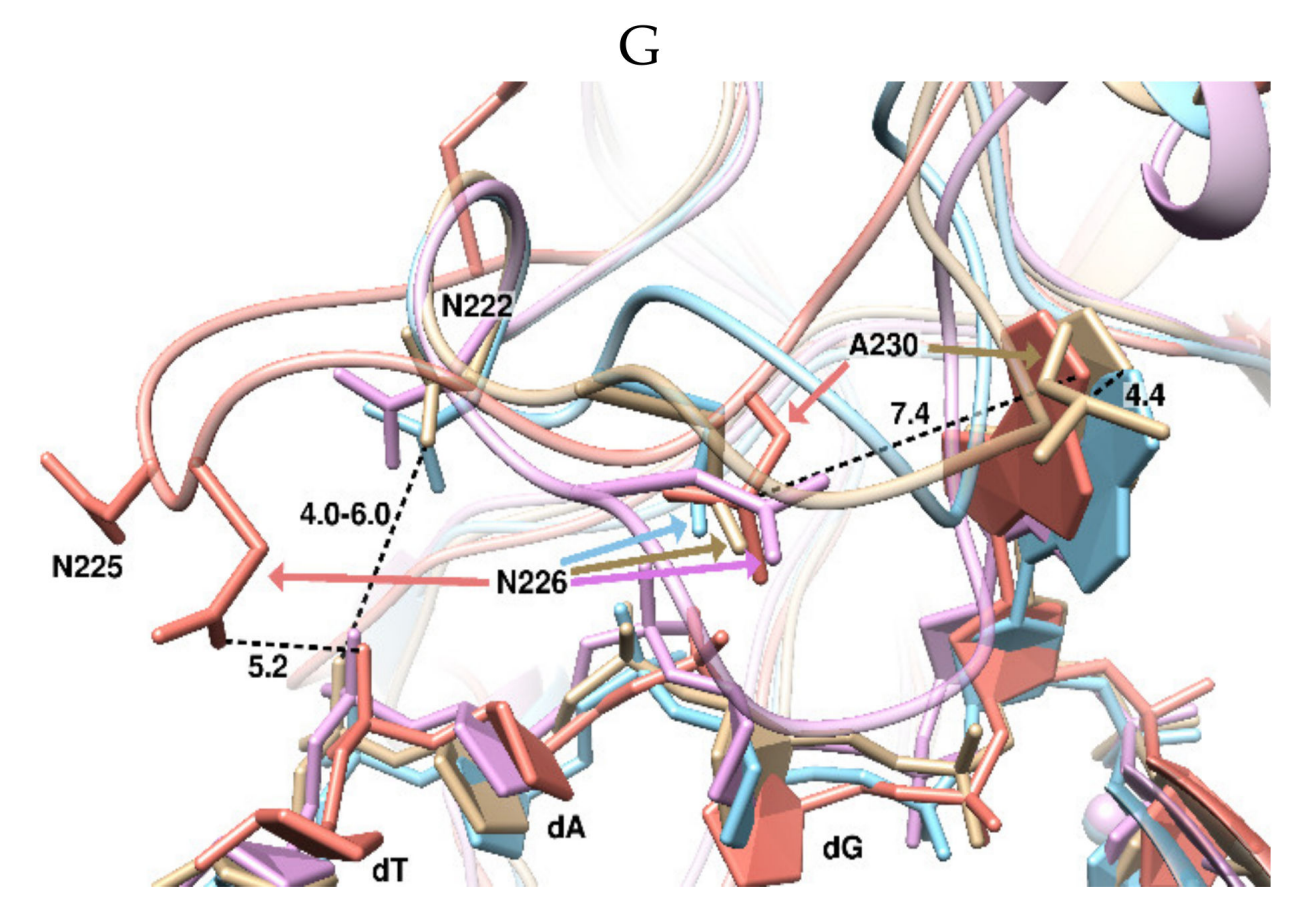
MD trajectories and structures of the complexes of APE1-like endonucleases with εA-containing DNA (hAPE1 is sand-colored, zAPE1 is cyan, xAPE1 is magenta, and Rrp1 is red): (**A**) Structural models of enzyme–substrate complexes and average lengths of H-bonds. The average lengths of H-bonds His309 N^ε2^–εA O5′ and Asn212 N^δ2^–εA O5′ are indicated. The Gly231 O–Ala214 N distance was at least 5.2 Å among the four models; changes in (**B**) αA O5′–Asn212 N^δ2^, (**C**) αA O5′–Asp210 O^δ1^, (**D**) αA O5′–His309 N^ε2^, (**E**) Gly231 O–Ala214 N, and (**F**) Thr233 O^γ1^–Glu236 O^ε1^ distances throughout the MD trajectories of the investigated APE1-like enzymes in complex with εA-containing DNA; (**G**) the structure of the Asn222–Ala230 loop in the models involving εA.

All four models of the complexes of enzymes with the εA-containing DNA manifested similar positions of the εA base in the active-site pocket, which were also similar to the positions of αA in the complexes involving hAPE1, zAPE1, and xAPE1 (Figure 9A).

By contrast, the analyses of the MD trajectories of the complexes with εA-containing DNA revealed the highest variability in the average distances between the atoms of the damaged base and residues of the active site, in comparison with the other damaged DNAs.

In the initial parts of the trajectories, there were no water molecules inside the active sites, and the average distance between εA O5′ and Asn212 N^δ2^ varied from 3.0 to 3.1 Å in zAPE1 and Rrp1 (which is 0.1 Å less than that in complexes with the F-site) to 3.4–3.5 Å in hAPE1 and xAPE1; the latter distance was slightly greater than that in F-site complexes (Figure 9B). Next, this distance jumped to >4.0 Å in the trajectories involving hAPE1 and zAPE1 because the incoming water molecule disrupted the direct interaction between εA O5′ and Asn212 N^δ2^. The distance between εA O5′ and Asp210 O^δ1^ was quite stable, too, and similar to that of the F-site complexes (4.4 Å on average; Figure 9C). This distance was affected by a water molecule only for hAPE1, resulting in an increase in distance up to 6.0 Å.

The distance between εA O5′ and His309 N^ε2^ was relatively stable in the initial parts of the trajectories involving hAPE1 (3.1 Å on average) and xAPE1 (3.3 Å on average); however, in the other two complexes, it varied significantly (from 3.5 to 3.9 Å), and these changes did not correlate with the movement of the water molecule in the active site (Figure 9D). Nevertheless, the water molecule then caused jumps of this distance in the zAPE1 and xAPE1 models.

As in the case of αA, the complex of Rrp1 with εA-containing DNA was the only one that remained unaffected by a water molecule entering the active site. On the other hand, the H-bonds Gly231 O–Ala214 N and Thr233 O^γ1^–Glu236 O^ε1^ broke up in all four complexes with εA (Figure 9E,F). In addition, the loop containing residues Asn222–Ala230 in the complexes with εA-containing DNA changed dramatically relative to its position in all the previous models. The contact of N226 and N225/N222 with DNA phosphates was very weak, and the corresponding distances constantly varied between 4.0 and 6.0 Å in all the trajectories (Figure 9G). Moreover, it was found that Ala230, which was the closest residue to the εA base at the start of every trajectory, shifted further from the base by 3.0 Å in the case of Rrp1, compared with the complex of hAPE1 with εA-containing DNA. To demonstrate the shift of the loop, the distances between εA C11 and Ala230 C^α^ in the models involving hAPE1 (4.4 Å) and Rrp1 (7.4 Å) are depicted in Figure 9G. The shift of this loop in Rrp1 eliminated all contact with dA.

## 3. Conclusions

Although the catalytic domain of the analyzed enzymes was mostly conserved, with stand-alone substitutions of some functionally important amino acid residues, it was reported [33] that these enzymes possess different abilities to incise damaged DNA containing one of the four types of damaged nucleotides: the F-site, DHU, αA, and εA. An intriguing question is that of why Rrp1 cannot cleave an αA-containing DNA, in contrast to hAPE1, zAPE1, and xAPE1. The damage-recognition loop of APE1-like enzymes containing residues 229–233 (the numbering corresponds to hAPE1) has to be moved out to enable the eversion of the damaged base; recently reported data [29] on the plasticity of the active site of hAPE1 allow us to suggest that this loop is a hindrance for the flipping of some damaged bases in homologous enzymes.

Therefore, in the present study, we performed a comparative MD simulation analysis of complexes of the four APE1-like enzymes with damaged DNA. Our MD findings elucidated the contact between the amino acid residues of the active site (of each homologous APE1-like enzyme) and the analyzed set of damaged DNA substrates. It was found that every damaged nucleotide had its unique position in the active site and a specific set of interactions with the nearest amino acid residues.

As expected, hAPE1 and zAPE1, which do not have any major differences in the active site and its vicinity, manifested similar locations of damaged nucleotides in the catalytic pocket. Despite two substitutions in xAPE1 (N229T and A230P), this enzyme did not manifest differences from hAPE1 and zAPE1 in the positions of the analyzed damaged DNAs.

On the other hand, Rrp1 showed the most pronounced deviations in its structure relative to hAPE1, zAPE1, and xAPE1. We noticed that the complexes between Rrp1 and the F-site- or DHU-containing DNA did not have any specific features, but in the complexes with bulkier αA- and εA-containing DNA, the observed differences were significant. For instance, in the complex of Rrp1 with εA-containing DNA, the average distances between the phosphate group and catalytic amino acids were acceptable for catalysis, but the damage-recognition loop Asn222–Ala230 was significantly shifted and disrupted; this suggests that this loop ensures the plasticity of the active-site pocket in the case of bulky lesions. By contrast, in the complex of Rrp1 with αA-containing DNA, the DNA backbone did not assume the same position as it had in the complexes with hAPE1, zAPE1, or xAPE1, and the distances to catalytic residues were not short enough in comparison with the other cases, thereby resulting in the complete loss of Rrp1 cleavage activity toward an αA-containing DNA. These findings suggest that substitutions of some residues in the damage-recognition loop (positions 222–230) may significantly affect catalytic-complex formation. Indeed, our data indicate that single substitution G225N in Rrp1 gives rise to direct contact between Asn225 and the phosphate group of dA situated on the 3′ side of αA. Consequently, the emergence of an H-bond between Asn225 and damaged DNA may be one of the major discriminatory factors in the course of catalytic-complex formation by APE1-like enzymes.

Still, there is a question about the exact stage of catalysis in which the steric hindrances described above play the main role. To find the answer to this question, simulations of the complete flipping-out pathway of the damaged nucleotide are required. We suggest that such an approach will allow researchers to elucidate the moment when every damaged base clashes with the protein loops. The snapshots of the flipping-out trajectory can help to define the peculiarities of early protein-damage interactions in cases of different damaged nucleotides, which could be important factors in controlling substrate specificity.

## 4. Materials and Methods

### MD Simulations

As a source of initial structure for the hAPE1 model, we used the crystal structure of this protein bound to a DNA product containing the F-site (PDB ID 4IEM) [18], which was manually converted to an initial uncleaved substrate. Additionally, an A-T pair was added to the end of the DNA duplex on the 5′ side of the damage to reduce edge effects on DNA binding.

To construct the initial structure of an enzyme–substrate complex for zAPE1, we utilized the crystal structure PDB ID 2O3C [35], with a free protein and an added DNA duplex and Mg^2+^ ions via the alignment of the protein part with hAPE1 structure (PDB ID 4IEM).

The crystal structures of xAPE1 from *X. laevis* and Rrp1 from *D. melanogaster* are currently unknown. Therefore, to obtain their 3D structures, we used the homology-modeling service Swiss-model [36]. The N-terminal domains of the enzymes were not taken into account during the homology modeling. The sequence of xAPE1 was cut to attain the same length as in hAPE1, and started from Val42; Rrp1 was truncated, too, and started from Lys423, which is the analog of Lys58 in hAPE1. The DNA duplex and Mg^2+^ were added to these structures in the same way as for zAPE1.

All nucleotides containing a damaged base were introduced into DNA through substitution of the F-site and positioned as described in ref. [31].

The simulations were performed in GROMACS 2019.6 [37] with the AMBER ff99SB-ILDN force field [38]. Hydrogen atoms were placed onto the structure by means of pdb2gmx. The solvent was represented by TIP3P [39] water molecules in periodic dodecahedron boxes, with at least a 1 nm layer of the solvent between a protein–DNA complex and the boundaries. Certain solvent molecules were replaced with counterions to neutralize the system. Simulations were performed under 300 K with protonated histidine residues. The Verlet cutoff scheme [40] was chosen with a cutoff of 1.2 nm for both van der Waals and electrostatic interactions, whereas H-bonds were constrained using the LINCS method [40]. Electrostatic interactions were computed in PME [41]. The solvated systems were minimized through steepest-descent minimization. After that, the systems were equilibrated in two stages, with restraints on DNA and protein heavy atoms: for 200 ps in the NVT ensemble, and for a subsequent 200 ps in the NPT ensemble. Productive dynamics were implemented with a 2 fs time step in the NPT ensemble, and the coordinates were saved every 10 ps. The length of every trajectory was at least 100 ns. To prevent DNA melting at the ends of the duplex, 8 kJ/(mol·nm^2^) restraints were applied to the atoms of the terminal nucleotides [42].

## Data Availability

Data are available upon request to N.A.K. Tel. +7-383-363-5174, E-mail: nikita.kuznetsov@niboch.nsc.ru.

## Abbreviations

AP site: apurinic/apyrimidinic site; APE1: AP endonuclease 1; NIR: nucleotide incision repair.
